# Impact of bulk density assignment of bone on MRI‐based abdominal region radiotherapy planning for MR‐linac workflow

**DOI:** 10.1002/acm2.70059

**Published:** 2025-02-25

**Authors:** Kota Abe, Masato Tsuneda, Yukio Fujita, Yukinao Abe, Takashi Uno

**Affiliations:** ^1^ Department of Radiation Oncology MR Linac ART Division Graduate School of Medicine Chiba University Chiba Japan; ^2^ Department of Radiological Sciences Komazawa University Tokyo Japan; ^3^ Department of Radiology Chiba University Hospital Chiba Japan; ^4^ Diagnostic Radiology and Radiation Oncology Graduate School of Medicine Chiba University Chiba Japan

**Keywords:** abdominal region, bulk density‐based treatment planning, MR‐linac, radiotherapy

## Abstract

**Purpose:**

The purpose of the present study was to evaluate the impact of bone relative electron density (rED) assignment on radiotherapy planning for the abdominal region.

**Methods:**

Twenty patients who received abdominal radiotherapy using MR‐Linac and underwent magnetic resonance imaging (MRI) and computed tomography (CT) simulation were analyzed. The reference plan (RP) was established using both CT and MR image sets (RP_CT and RP_MRI). The RP_MRI utilized the bulk density method. The recalculated RPs derived from various rED assignment methods were evaluated for comparison on both datasets. The RPs were recalculated by excluding rED assignment for bones (scenario A). Based on the International Commission on Radiation Units and Measurements report, lung contours were assigned rED of 0.258, and body contours were assigned 1.000 (scenario B) and 1.019 (scenario C). Dose volume histogram (DVH) differences between the three recalculated scenarios and RPs were evaluated. D95, D99, and D1cc were evaluated for target volumes, including gross tumor volume, internal target volume, and planning target volume. DVH parameters, including D1cc for each abdominal organ at risk (OAR) and the mean dose to the liver and kidneys, were evaluated. Three‐dimensional local gamma analysis was conducted to assess dose distribution differences between the three recalculated scenarios and RPs.

**Results:**

In all scenarios of the CT‐ and MRI‐based validation, the average gamma pass rates (2%/2 mm) were higher than 95%. In the CT‐based validation, all target DVHs across the 20 patients showed that none exceeded 2% error in scenario A, whereas 2% and 14% exceeded the threshold in scenarios B and C, respectively. For OARs in CT and MRI‐based validation, absolute maximum dose differences when compared with those of the RP were 0.19 Gy and 0.22 Gy, respectively, in scenario A.

**Conclusion:**

Excluding bone rED considerations in abdominal treatment planning may not yield notable clinical differences.

## 1 INTRODUCTION

In recent years, clinical practice has increasingly adopted radiotherapy systems capable of online adaptive radiotherapy (ART).^1^, ^2^ The MR‐Linac, which combines a magnetic resonance imaging (MRI) system with a radiotherapy system, enables online ART using MR images.^3^ MRI on the day of treatment is performed at each treatment session. To enhance the accuracy of deformable image registration (DIR), it has been proposed that MR images can be used as planning images for reference treatment plans, utilizing the same modality for both reference and treatment‐day image acquisition.^4^ However, MR images do not contain electron density information. Therefore, to create treatment planning using MR images, it is necessary to create synthetic CT images. MRCAT is a commercially available software that generates synthetic CT images from MR images, and its clinical implementation is also being advanced.^5^ However, there are problems regarding the cost of implementing these solutions, and their functionality may not be compatible with the abdominal region. Otherwise, the organs are delineated on the MR image, and the bulk density method is often used to assign electron density information uniformly to the delineated contours.^6^, ^7^ The treatment workflow of Elekta Unity (Elekta AB, Stockholm, Sweden) implements the bulk density method to generate synthetic CT images and achieve adaptation workflow using MR images.^3^ Several treatment planning methods using the International Commission on Radiation Units and Measurements (ICRU)‐based and population‐based bulk density methods have been developed^8^, ^9^, ^10^, ^11^; however, both methods require delineation of the organ to which the relative electron density (rED) is to be assigned. Lee et al. compared the accuracy of dose calculations in radiotherapy planning for prostate cancer patients with and without bone rED assignment based on uniform water replacement in the body, using CT image‐based calculations as a reference.^11^ The dose difference was greater than 2% when comparing the reference condition to the calculated results without bone rED assignment. A dose difference of up to 12% was observed in some patients. The prostate is surrounded by pelvic bones. Therefore, bone rED assignment has a significant effect on dose calculation. In contrast, the abdominal region is surrounded by ribs, which are less likely to contribute to radiation attenuation owing to their smaller bone thickness. Additionally, less than half of the radiation beams utilized in treating tumors in the liver, kidney, and pancreas pass through the spine. Thus, the effect of bone rED assignment on the dose‐volume histogram (DVH) parameters of the abdominal organs at risk (OAR) may be smaller than that for the pelvic site. Bello et al. reported that using the bulk density method, it required approximately 17.5 min to create contours and approximately 1 min to generate the rED map.^12^ Additionally, manually contouring of ribs adds a significant clinical burden because of their frequently insufficient delineation when the thresholding process is applied to images. Hsu et al. evaluated the effect of the bulk density method in MRI‐based radiotherapy treatment planning for the abdomen.^10^ When compared with calculations based on CT images, a mean dose difference of approximately −3.5% was observed in PTVD99.9 for patients with liver tumors near the lungs. This difference was observed when performing dose calculations in the abdominal area, assuming the entire body contour's rED as water. Mean relative dose differences of −0.4% and −0.5% were also observed for the population‐based and ICRU‐based bulk density methods with three rED assignments that included water, lung, and bone, respectively. However, no previous studies have evaluated the effect of rib or spinal bone rED assignment on treatment planning in the abdominal region. Furthermore, the secondary electrons under a magnetic field are deflected by the Lorentz force, which affects the dose distribution.^13^ Therefore, in regions with inhomogeneous densities, such as the boundary between the liver and lung, there may be an influence from the electron return effect. Consequently, in addition to the DVH parameter, it is necessary to evaluate spatial dose differences using gamma analysis. Rippke et al. evaluated the effectiveness of the bulk density method for abdominal MR‐guided radiotherapy.^14^ However, since their study evaluated a synthetic CT generation method that included bone rED assignment, it remains unclear how the with or without of rib or spinal bone rED assignment affects the dose distribution in the presence of a magnetic field.

Delineation of the ribs or spinal bone in the abdominal region may be unnecessary during radiotherapy planning using the bulk density method if the impact of bone rED assignment on the DVH and dose distributions is clinically acceptable. This could increase the efficiency of the reference treatment planning and simplify online adaptive radiotherapy in the MR‐Linac workflow. Therefore, this study aimed to evaluate the impact of bone rED assignment on the DVH and dose distributions in radiotherapy planning for the abdominal region and to assess the feasibility of an ICRU‐based rED assignment method.

## 2 METHODS

### 2.1 Patients

Twenty patients who underwent radiotherapy of the abdominal region using the MR‐Linac system at our institution were included in the analysis. Table 1 presents the patient characteristics. The study population comprised ten, five, and five patients with liver, pancreatic, and kidney cancers, respectively. All patients underwent stereotactic body radiotherapy (SBRT) at 35–50 Gy in five or eight fractions. In addition, all patients underwent simulation CT acquisition on the same day as MRI acquisition. CT images were acquired to obtain rED information using Aquilion LB (Canon Medical Systems, Otawara, Japan) at 120 kV with a slice thickness of 2 mm.

**TABLE 1:** Patient characteristics.

Patient Characteristic		Overall *n* = 20
Gender	Female	8
	Male	12
Age (years: mean ± SD)		67.6 ± 13.1
Treatment sites	Liver	10
	Kidney	5
	Pancreas	5
Tumor location of liver patients	S2	1
	S4	2
	S6	3
	S7	1
	S7/8	2
	S8	1
Number of beams during IMRT	Liver	9
	Kidney	9―11
	Pancreas	13
Total treatment dose (fraction)	Liver	35―50 (5)
	Kidney	35―40 (5)
	Pancreas	40 (5―8) (unknown: n = 5)
Cases with PTV adjacent to Bone	Liver	6
	Kidney	3
	Pancreas	4
Cases with PTV adjacent to Lungs	Liver	3
	Kidney	2
	Pancreas	0

Abbreviations: IMRT, intensity modulated radiotherapy; PTV, planning target volume.

### 2.2 Contouring

The organ contours were delineated on the MR and CT images of each patient, and the average rED value for each organ was calculated from the CT images. The delineation of organ contours on CT images was performed using free‐breath CT. For the dose calculation on MRI, these values were subsequently assigned to the same organ on the MR images for each patient. Body, bones, and gross tumor volume (GTV) contours were delineated on the images of all patients; however, normal tissues such as the bowel, colon, duodenum, stomach, kidney, and liver were minimally delineated if located within ±1 cm of the PTV in the SI direction, while organs intersected by the treatment beam were delineated even if distant from the PTV. The internal target volume (ITV) margins containing tumor motion were added to the GTV using four‐dimensional CT (4DCT) images. Furthermore, the planning target volume (PTV) was created by adding a 5‐mm margin to the ITV. Air pockets present in the OARs were included in the organ contours. The spine and ribs were delineated as a single structure: “Bone.” Body contours on the CT images were created via a thresholding process on the RayStation treatment planning system (TPS) version 10A SP1 (RaySearch Laboratories AB, Stockholm, Sweden) using Hounsfield unit (HU) values. The bone contours on the CT images were drawn via atlas‐based segmentation and modified manually as necessary. The body and bone contours on the MR images were propagated from the CT images to the MR images using the deformable image registration of the Adapt Anatomy function in Monaco v. 5.51.11 (Elekta AB, Stockholm, Sweden). Contour corrections were performed manually, if necessary. The rED value of the body contour was calculated after subtracting the area of other contoured organs.

### 2.3 Treatment planning

The CT‐ED table was obtained using a Model 467 electron density phantom (Gammex Inc., Middleton, WI, USA). The tumor was set as the image center in the superior‐interior (SI) direction on the MR images acquired for treatment planning. The SI image range was set as ±14 cm from the center of the tumor. The reference plans (RP) for all patients were created using MR and CT images. Treatment plans were created using the step‐and‐shoot intensity‐modulated radiotherapy (IMRT) method with 9–13 beams. Dose calculations were performed using the Monaco TPS, and included the effect of the magnetic field on dosimetry. Dose calculations for all treatment plans were performed assuming a Monte Carlo uncertainty of 1% per calculation and dose calculation grid of 2.5 mm. For the MRI dose calculation, the bone, body, abdominal organ, and GTV were delineated on the MR images, and the rED values obtained from the CT images were assigned to each patient. The rED values assigned to each organ in the MR images were calculated from the CT images of each patient using Monaco TPS. The plan created based on MR images using this method is hereafter referred to as the reference plan (RP_MRI). For the dose calculation on CT images, voxel‐based rED information was used, as in routine clinical practice. By examining the CT images, it is possible to evaluate the impact of bone rED assignment while minimizing the uncertainty associated with using the RP created with bulk density assignment for MR image validation. The plan created on CT images using this method is hereafter referred to as the reference plan (RP_CT).

### 2.4 rED assignment and dose recalculation

**FIGURE 1:**
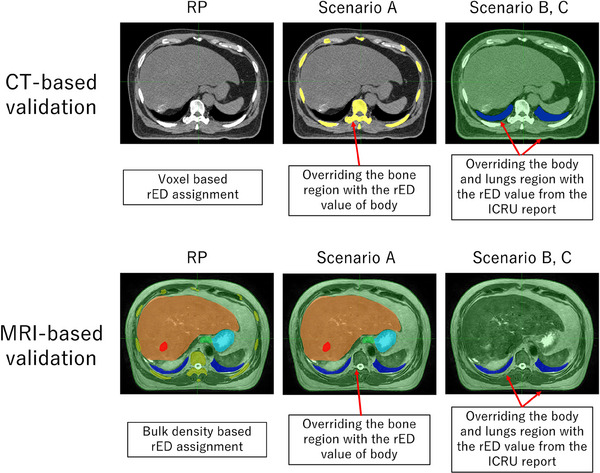
Distribution map of rED in the reference plan (RP), Scenario A, and ICRU‐based assignment (Scenarios B and C). In the RP, rED was assigned to the rib and spinal bones, whereas in Scenario A and the ICRU‐based assignment, it was not assigned. In other words, in Scenario A bone region was overridden by the rED value of each patient's body. Furthermore, in the ICRU‐based assignment, all internal organs, excluding the lungs, were uniformly set to rED =  1.000 (Scenario B) and 1.019 (Scenario C).

This study evaluated the DVH differences compared with RP_CT and RP_MRI under scenarios A, B, and C, as described below. Figure 1 presents examples of rED assignments for the RP and each scenario. When performing bulk density assignment on the CT dataset, the average rED value on each CT image for each contour was uniformly assigned to the contour using the force rED function.

- The bone rED assignment was excluded in the RP settings and the bone rED was overwritten with a body rED for dose calculation.
- The body was divided into two structures: the lung region and the body contour without the lungs. Dose calculation was performed by assigning the respective rED values from ICRU report 46^15^ to the lung and water rED. The lung region was assigned a rED value of 0.258, whereas the body contour without lungs was assigned a rED value of 1.000.
- Similar to scenario B, except that the rED of the body was assumed to be soft tissue instead of water, assigning a rED of 1.019, which is the mean value of the average soft tissue for adult men and women in the ICRU report.

In the analysis using RP_CT, we conducted two additional scenarios: “Scenario_bulkCT,” which assigns the average rED value to each contour using bulk density method as in RP_MRI, and “ScenarioA_bulkCT,” which overwrites only the bone rED with the body rED from “Scenario_bulkCT.” The MU values of RP_CT and RP_MRI were fixed and the doses were recalculated under the above three scenarios. The DVH parameters evaluated for the target contours were D95 for the GTV, ITV, and PTV, and D99 and D1cc for the PTV. The DVH parameters used clinically at our institution were D1cc for normal tissue PRVs (2‐mm margin), including the colon, bowel, 　stomach, and duodenum, and the mean doses (Dmean) for the liver and kidney were also evaluated. Subgroup analysis was conducted for cases in which the tumors were close to the bone or lung (within 1 cm) to investigate whether the inhomogeneity of densities caused by the proximity of the bone and lung regions under magnetic fields led to deviations from the RPs. Additionally, plans with a higher proportion of beams passing through the spinal bones were analyzed to determine the extent of the spinal bone's influence on the dose difference. The “pass through” beam was defined as a beam that intersected the spinal bone before reaching the PTV. In the process of calculating the rED values of the body from CT images, rED values were obtained from body contouring, excluding bones. Implementing a workflow that ignores the rED assignment for bones can result in rED calculations using body contours, including bones, owing to the omission of an explicit bone structure delineation. Therefore, we also examined whether dose calculations based on the rED value of body contouring that incorporates bones deviate significantly from RP_MRI.

### 2.5 Analysis

Three‐dimensional (3D) local gamma analysis was conducted using a 3D slicer software to assess the overall dose distribution.^16^ Evaluations of the dose difference and distance to agreement were performed at settings of 2%/2 mm using a 10% dose threshold. For the comparison of DVH parameters in this study, the acceptance criteria for dose differences from the RP were less than 2.0%. To demonstrate the reliability of the calculation method used in this study, we analyzed a patient with spinal metastasis. In this case, the PTV contour was used as a mask and gamma analysis was performed within that region. In this case, significant discrepancies in the targets and OAR DVH parameters could have occurred if rED assignment to the bone was not performed.

## 3 RESULTS

### 3.1 rED acquisition

The mean bone and lung rED of the patients included in the present study were 1.154 ± 0.029 and 0.291 ± 0.058, respectively. The mean rEDs values of the colon, bowel, duodenum, stomach, R_kidney, L_kidney, and liver obtained from CT images of each patient were 0.840 ± 0.010, 0.957 ± 0.034, 0.983 ± 0.101, 0.928 ± 0.105, 1.031 ± 0.011, 1.030 ± 0.009, and 1.054 ± 0.010, respectively.

### 3.2 Evaluation of the dose distributions with 3D gamma analysis and DVH parameters

**FIGURE 2:**
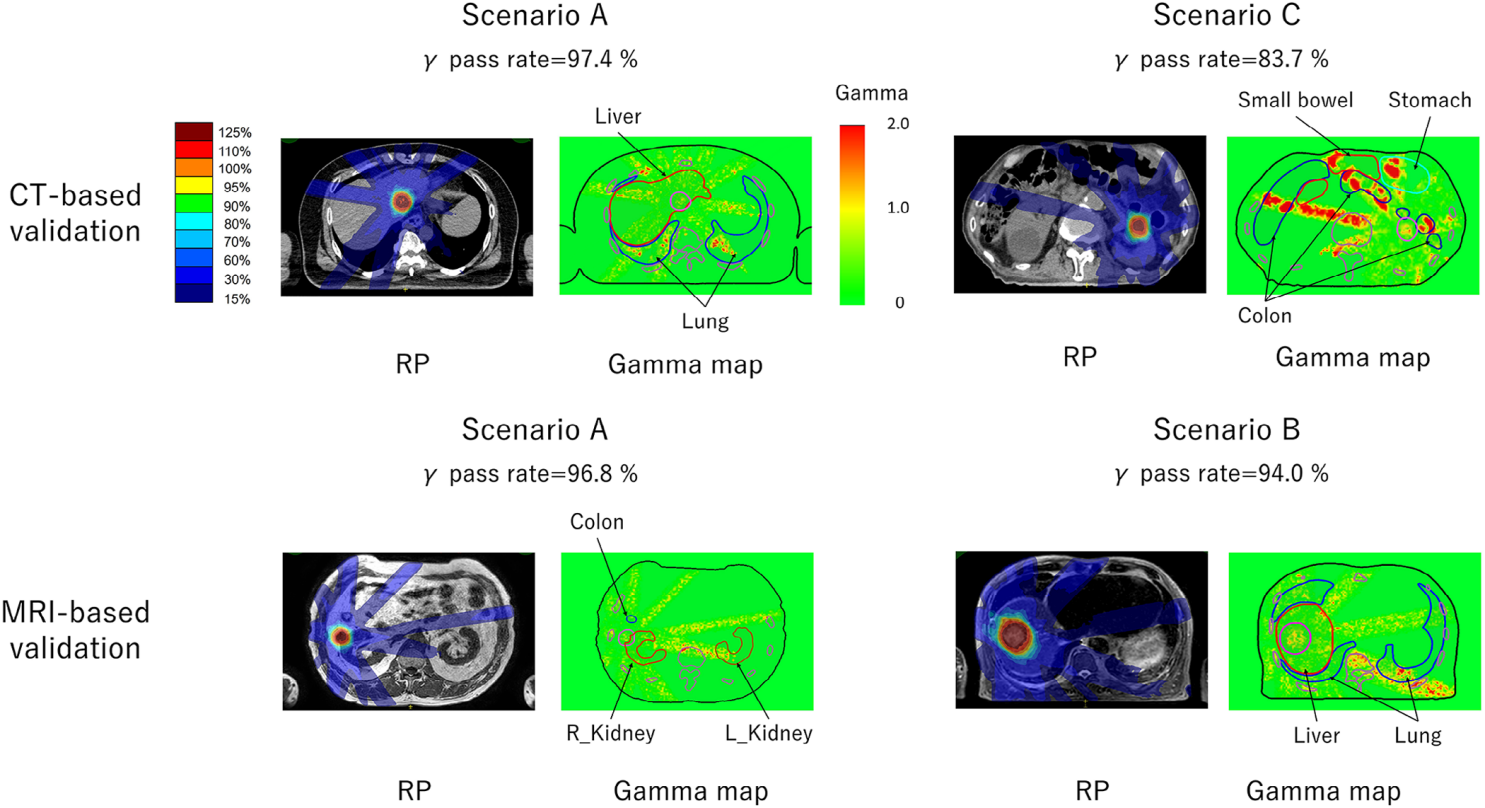
Worst cases of γ analysis (2%/2 mm) on CT and MRI‐based validation. In the CT‐based‐ and MRI‐based validations, the worst cases for Scenario A and for both Scenarios B and C are shown.

**FIGURE 3:**
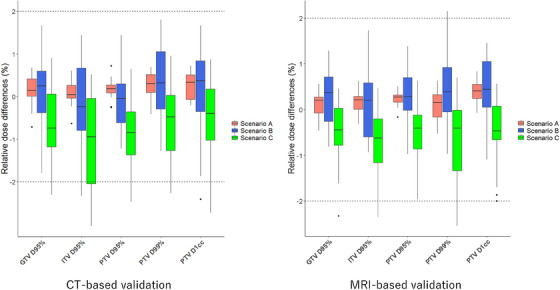
Boxplot of dose differences in the target contours when comparing the reference plan (RP) to the recalculated results for each scenario on the CT and MRI datasets. The dotted line represents a difference of 2.0%, which was set as clinically significant. Scenario A represents recalculations without assigning the rED of the bones in each RP setting. Scenario B represents the recalculations of the RP, with the rED of the entire body and lungs set to 1.000 and 0.258, respectively. Scenario C represents the recalculations of the RP, with the rED of the entire body and lungs set to 1.019 and 0.258, respectively.

**FIGURE 4:**
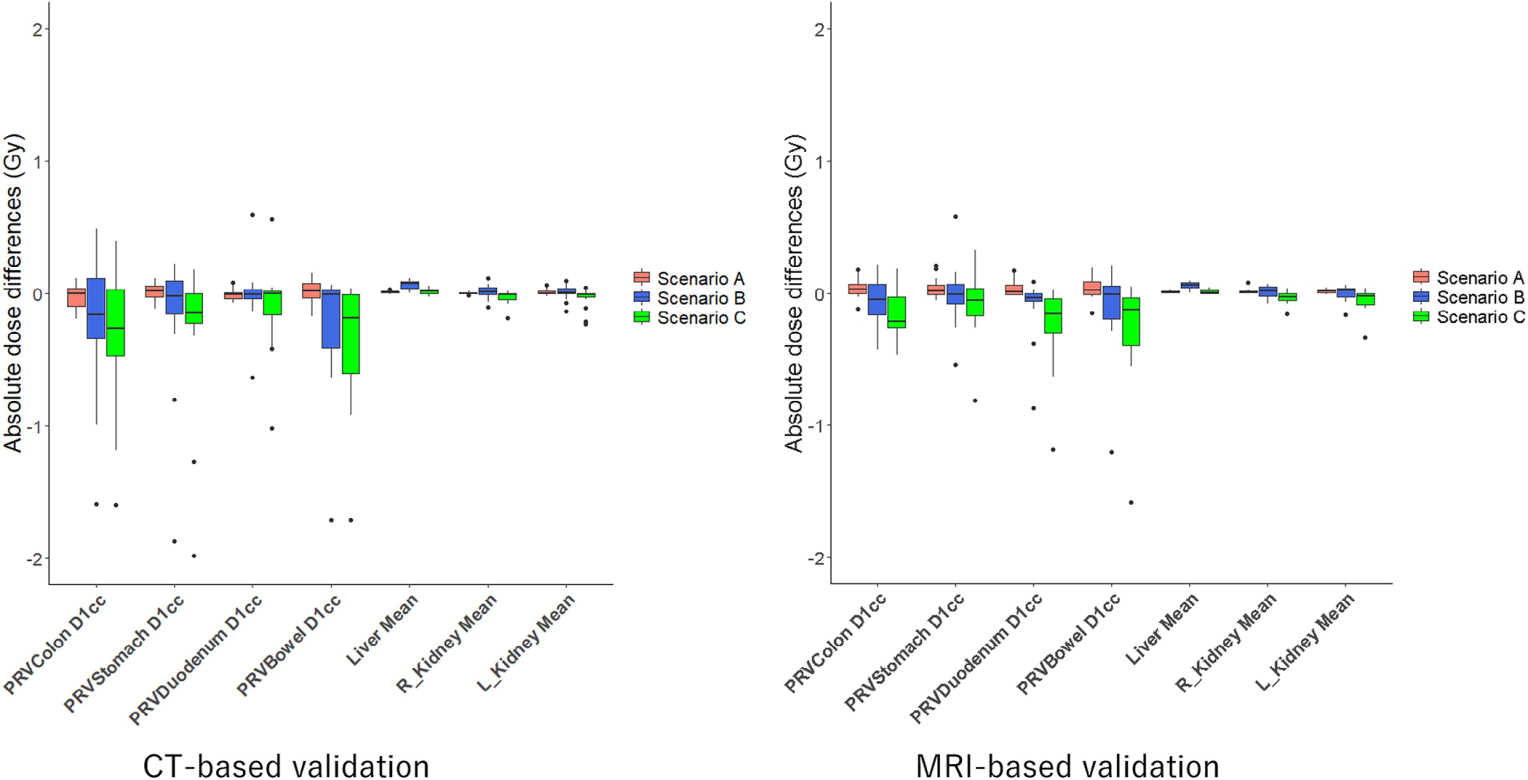
Boxplot of absolute dose differences in the OARs when comparing the reference plan to the recalculated results for each scenario on the CT and MRI datasets. Scenario A represents recalculations without assigning the rED of the bones in each RP setting. Scenario B represents the recalculations of the RP, with the rED of the entire body and lungs set to 1.000 and 0.258, respectively. Scenario C represents the recalculations of the RP, with the rED of the entire body and lungs set to 1.019 and 0.258, respectively.

Table 2 shows the local 3D gamma analysis results at the 2%/2 mm criteria for each scenario compared to RP_CT and RP_MRI. The worst cases for gamma analysis of 2%/2 mm on CT and MRI datasets in scenario A and scenario B or C are shown in Figure 2. As shown in Figure 2, for Scenario A, even in the worst case, the gamma pass rate exceeded 96%. Additionally, gamma analysis failures in the lower dose region (below the 30% isodose line) were more noticeable than those around the PTV. For scenario C, in the CT‐based validation, the lowest gamma pass rate (83.7%) was observed in cases with significant bowel gas. When comparing voxel‐based and bulk density dose calculations in the CT dataset (Scenario_bulkCT), the absolute mean dose differences for the target and 3D gamma analysis (2%/2 mm) were 0.51% and 96.3%, respectively. In contrast, in ScenarioA_bulkCT, the absolute mean dose differences for the target and 3D gamma analysis (2%/2 mm) were 0.29% and 96.0%, respectively. The DVH parameters in the three scenarios were evaluated and compared with those of RPs. Figures 3 and 4 show the results for the target and OARs on CT and MRI‐based validation, respectively. For the target DVH, Tables 3 and 4 indicate the average percentage differences and their range for each scenario compared with the RP_CT and RP_MRI and further present the results of the subgroup analysis. The subgroup analysis showed no clear differences from the overall analysis. In Table 5, for OARs, the average differences and their range for each scenario when compared with RP_CT and RP_MRI are presented in terms of both percentage and Gy. The mean dose differences in the five DVHs according to the target volume in the CT‐based validation were 0.20%, 0.09%, and ‐0.77% for scenarios A, B, and C, respectively. For the MRI‐based validation, they were 0.21%, 0.27%, and ‐0.57% for scenarios A, B, and C, respectively. For OARs in CT‐based validation, the mean dose differences compared with those of the RP were 0.13%, −0.37%, and −1.01% for scenarios A, B, and C, respectively, and in MRI‐based validation, they were 0.17%, −0.01%, and −0.72% for scenarios A, B, and C, respectively. Among the 100 DVHs evaluated for 20 patients across the five target DVH parameters in the CT‐based validation, it was observed that the number of DVH parameters with errors exceeding 2.0% was zero (0%) for scenario A, two (2%) for scenario B, and 14 (14%) for scenario C. For the MRI‐based validation, they were zero (0%), one (1.0%), and four (4.0%) for scenarios A, B, and C, respectively. Furthermore, the maximum absolute dose differences for OAR DVHs were 0.19 Gy in scenario A, 1.87 Gy in scenario B, and 1.98 Gy in scenario C. For MRI‐based validation, they were 0.22 Gy, 1.21 Gy, and 1.58 Gy for scenarios A, B, and C, respectively. In the 20 patients included in this study, the average rED of the body was 0.994 ± 0.014 when including bone structures, and 0.980 ± 0.013 when excluding bones from the body contour. The mean dose difference between plans calculated with body rED that included bone and plans calculated with body rED that did not include bone (RP_MRI) was −0.04% and −0.03% for the target and OAR DVH indices, respectively. The results of analyzing the SBRT treatment plan for patients with spinal metastasis using the same methods as in scenario A are presented in Figure S1 and Table S1 in the Supplemental materials. The DVH differences were more pronounced than those of the abdominal organ cases, and the gamma map indicated considerable errors within the PTV.

**TABLE 2:** The local 3D gamma analysis results at the 2%/2 mm criteria for each scenario compared to the reference plan established using CT image (CT‐based validation) and MRI (MRI‐based validation).

	Local 3D gamma pass rate [%]	
	CT‐based validation	MRI‐based validation
Scenario A (20 cases)	98.7 ± 0.74	98.6 ± 0.78
Scenario B (20 cases)	95.5 ± 3.39	98.0 ± 1.27
Scenario C (20 cases)	95.2 ± 3.55	97.6 ± 1.26
Scenario A: Cases with PTV adjacent to Bone (13 cases)	98.7 ± 0.79	98.4 ± 0.91
Scenario B: Cases with PTV adjacent to Bone (13 cases)	95.8 ± 2.74	97.7 ± 1.51
Scenario C: Cases with PTV adjacent to Bone (13 cases)	95.5 ± 2.47	97.4 ± 1.43
Scenario A: Cases with PTV adjacent to Lungs (5 cases)	98.2 ± 0.92	97.9 ± 0.72
Scenario B: Cases with PTV adjacent to Lungs (5 cases)	94.0 ± 3.38	96.9 ± 1.86
Scenario C: Cases with PTV adjacent to Lungs (5 cases)	94.1 ± 2.67	97.1 ± 1.02
Scenario A: Cases with beam passing through spinal bone to PTV (16 cases)	98.6 ± 0.77	98.7 ± 0.61
Scenario B: Cases with beam passing through spinal bone to PTV (16 cases)	95.2 ± 3.67	98.0 ± 1.26
Scenario C: Cases with beam passing through spinal bone to PTV (16 cases)	94.9 ± 3.86	97.7 ± 1.02

Abbreviations: 3D, three‐dimensional; PTV, planning target volume.

**TABLE 3:** Mean relative percentage differences and their range for each scenario compared to the reference plan established using CT image for target DVH parameters and the results of subgroup analysis.

	Relative percentage difference [range]				
Targets DVH parameters	GTV D95%	ITV D95%	PTV D95%	PTV D99%	PTV D1 cc
Scenario A (20 cases)	0.16 [−0.71−0.68]	0.10 [−0.63−0.61]	0.19 [−0.25−0.72]	0.28 [−0.42−0.70]	0.28 [−0.20−0.72]
Scenario B (20 cases)	0.19 [−1.79−1.66]	−0.13 [−2.33−1.45]	−0.06 [−1.22−1.44]	0.27 [‐1.27−1.81]	0.16 [−2.41−1.67]
Scenario C (20 cases)	−0.67 [−2.30−0.90]	−1.08 [−3.0−0.52]	−0.88 [‐2.47−0.65]	−0.61 [−2.27−0.95]	−0.60 [−2.72−0.88]
Scenario A: Cases with PTV adjacent to Bone (13 cases)	0.09 [−0.71−0.68]	0.07 [−0.63−0.61]	0.09 [‐0.25−0.32]	0.22 [−0.42−0.70]	0.27 [‐0.20−0.72]
Scenario B: Cases with PTV adjacent to Bone (13 cases)	0.18 [‐1.10−1.66]	−0.23 [−2.33−1.29]	−0.20 [‐1.20−1.26]	0.13 [−1.27−1.58]	0.29 [‐1.87−1.67]
Scenario C: Cases with PTV adjacent to Bone (13 cases)	−0.68 [−2.14−0.59]	−1.01 [−3.04−0.52]	−0.97 [‐2.47−0.65]	−0.75 [‐2.11−0.53]	−0.44 [−2.67−0.88]
Scenario A: Cases with PTV adjacent to Lungs (5 cases)	0.23 [‐0.03−0.68]	0.06 [−0.05−0.24]	0.19 [0.05−0.32]	0.21 [−0.01−0.41]	0.25 [‐0.08−0.66]
Scenario B: Cases with PTV adjacent to Lungs (5 cases)	0.61 [0.01−1.46]	−0.11 [−2.34−1.29]	0.09 [−0.65−1.26]	−0.07 [−1.27−1.20]	0.78 [0.22−1.67]
Scenario C: Cases with PTV adjacent to Lungs (5 cases)	−0.10 [−1.21−0.59]	−0.79 [−3.04−0.52]	−0.49 [−1.03−0.65]	−0.86 [−1.94−0.53]	0.06 [−0.69−0.88]
Scenario A: Cases with beam passing through spinal bone to PTV (16 cases)	0.26 [−0.20−0.68]	0.17 [−0.23−0.61]	0.22 [−0.25−0.72]	0.33 [−0.10−0.70]	0.35 [−0.17−0.72]
Scenario B: Cases with beam passing through spinal bone to PTV (16 cases)	0.30 [−1.79−1.66]	0.04 [−1.94−1.45]	0.07 [−1.22−1.44]	0.49 [−0.88−1.81]	0.19 [−2.41−1.67]
Scenario C: Cases with beam passing through spinal bone to PTV (16 cases)	−0.63 [−2.30−0.90]	−0.97 [−2.80−0.52]	−0.80 [−2.47−0.65]	−0.40 [−2.27−0.95]	−0.60 [−2.72−0.88]

Abbreviations: DVH, dose–volume histogram; GTV, gross tumor volume; ITV, internal target volume; PTV, planning target volume.

**TABLE 4:** Mean relative percentage differences and their range for each scenario compared to the reference plan established using MRI for target DVH parameters and the results of subgroup analysis.

	Relative percentage difference [range]				
Targets DVH parameters	GTV D95%	ITV D95%	PTV D95%	PTV D99%	PTV D1 cc
Scenario A (20 cases)	0.10 [−0.46−0.56]	0.17 [−0.31−0.62]	0.23 [−0.16−0.51]	0.12 [−0.53−0.62]	0.41 [−0.07−0.83]
Scenario B (20 cases)	0.27 [−0.81−1.28]	0.07 [−0.93−1.73]	0.25 [−0.97−1.38]	0.35 [−0.97−2.14]	0.38 [−1.09−1.45]
Scenario C (20 cases)	−0.50 [−2.32−0.45]	−0.71 [−2.35−0.47]	−0.55 [−1.96−0.63]	−0.64 [−2.54−0.70]	−0.43 [−2.00−0.56]
Scenario A: Cases with PTV adjacent to Bone (13 cases)	0.13 [−0.32−0.55]	0.22 [−0.14−0.62]	0.19 [‐0.16−0.41]	0.01 [‐0.53−0.56]	0.36 [−0.07−0.81]
Scenario B: Cases with PTV adjacent to Bone (13 cases)	0.26 [−0.81−1.28]	0.07 [−0.93−1.73]	0.26 [‐0.73−1.38]	0.30 [‐0.76−2.14]	0.36 [−0.97−1.45]
Scenario C: Cases with PTV adjacent to Bone (13 cases)	−0.47 [−2.32−0.45]	−0.62 [−2.35−0.47]	−0.49 [‐1.96−0.63]	−0.71 [−2.54−0.70]	−0.38 [−2.00−0.56]
Scenario A: Cases with PTV adjacent to Lungs (5cases)	0.11 [−0.26−0.33]	0.12 [−0.14−0.26]	0.21 [0.07−0.28]	0.03 [‐0.20−0.41]	0.36 [0.11−0.54]
Scenario B: Cases with PTV adjacent to Lungs (5 cases)	0.54 [−0.03−1.28]	0.30 [−0.53−0.78]	0.38 [‐0.05−0.81]	0.34 [0.10−0.79]	0.77 [0.16−1.45]
Scenario C: Cases with PTV adjacent to Lungs (5 cases)	−0.12 [−0.83−0.44]	−0.27 [−1.11−0.25]	−0.18 [‐0.76−0.35]	−0.23 [−1.05−0.42]	0.05 [‐0.51−0.56]
Scenario A: Cases with beam passing through spinal bone to PTV (16 cases)	0.11 [−0.46−0.56]	0.17 [−0.31−0.62]	0.24 [−0.16−0.51]	0.11 [−0.53−0.62]	0.47 [0.14−0.83]
Scenario B: Cases with beam passing through spinal bone to PTV (16 cases)	0.37 [−0.81−1.28]	0.13 [−0.93−1.73]	0.31 [‐0.97−1.38]	0.49 [−0.97−2.14]	0.44 [−1.09−1.45]
Scenario C: Cases with beam passing through spinal bone to PTV (16 cases)	−0.49 [−2.32−0.45]	−0.72 [−2.35−0.47]	−0.54 [‐1.96−0.63]	−0.59 [−2.54−0.70]	−0.42 [−2.00−0.56]

Abbreviations: DVH, dose–volume histogram; GTV, gross tumor volume; ITV, internal target volume; PTV, planning target volume.

**TABLE 5:** Mean differences and ranges of OAR DVH parameters compared to the reference plan established using CT image and MRI, presented as relative percentage and absolute dose (Gy).

OARs DVH parameters		Relative percentage difference[range] Dose difference[range]						
		PRVcolon D1 cc	PRVstomach D1 cc	PRVduodenum D1 cc	PRVbowel D1 cc	Liver Mean dose	R_Kidney Mean dose	L_Kidney Mean dose
CT	Scenario A	−0.35%[−2.15−0.56] −0.02 Gy[−0.19−0.11]	0.21%[−0.72−1.49] 0.02 Gy[−0.12−0.12]	0.11%[−0.57−2.11] −0.00 Gy[−0.07−0.09]	0.22%[−1.55−2.67] 0.02 Gy[−0.17−0.15]	0.16%[−0.09−0.39] 0.01 Gy[−0.00−0.04]	0.08%[−0.16−0.39] 0.00 Gy[−0.01−0.02]	0.47%[−0.19−1.81] 0.01 Gy[−0.02−0.07]
	Scenario B	−1.74%[−13.46−2.11] −0.22 Gy[−1.59−0.49]	−1.20%[−15.3−1.85] −0.17 Gy[−1.87−0.22]	−0.28%[−1.79−7.33] −0.01 Gy[−0.63−0.60]	−1.75%[−14.35−3.29] −0.25 Gy[−1.71−0.06]	0.99%[0.24−1.55] 0.07 Gy[0.01−0.12]	0.16%[−2.56−1.76] 0.02 Gy[−0.10−0.12]	0.67%[−1.33−4.37] 0.01 Gy[−0.13−0.09]
	Scenario C	−2.34%[−13.52−2.21] −0.33 Gy[−1.60−0.40]	−1.77%[−16.22−1.37] −0.26 Gy[−1.98−0.18]	−0.08%[−2.88−6.95] −0.11 Gy[−1.02−0.56]	−2.23%[−14.39−1.93] −0.37 Gy[−1.71−0.04]	0.20%[−0.42−0.74] 0.02 Gy[−0.02−0.06]	−0.58%[−3.18−0.34] −0.03 Gy[−0.19−0.02]	−0.26%[−2.10−2.80] −0.03 Gy[−0.23−0.05]
MRI	Scenario A	0.11%[−1.02−0.85] 0.04 Gy[−0.12−0.18]	0.18%[−1.43−0.78] 0.04 Gy[−0.05−0.21]	−0.03%[−1.93−0.55] 0.04 Gy[−0.02−0.17]	0.10%[−1.08−1.85] 0.03 Gy[−0.15−0.19]	0.14%[−0.09−0.30] 0.01 Gy[0.00−0.03]	0.29%[−0.02−0.92] 0.02 Gy[0.00−0.08]	0.38%[−0.11−1.64] 0.01 Gy[−0.01−0.04]
	Scenario B	−0.44%[−3.76−1.08] −0.06 Gy[−0.43−0.22]	−0.12%[−1.61−1.46] −0.01 Gy[−0.54−0.58]	−0.58%[−2.44−1.79] −0.12 Gy[−0.87−0.09]	−0.49%[−3.88−1.09] −0.12 Gy[−1.21−0.21]	0.84%[0.26−1.27] 0.06 Gy[0.01−0.09]	0.18%[−1.48−1.51] 0.01 Gy[−0.08−0.07]	0.52%[−1.25−4.21] 0.00 Gy[−0.16−0.06]
	Scenario C	−0.91%[−4.10−1.57] −0.17 Gy[−0.46−0.18]	−0.54%[−2.42−1.18] −0.08 Gy[−0.81−0.33]	−1.44%[−3.52−0.54] −0.27 Gy[−1.18−0.03]	−1.35%[−5.09−0.51] −0.28 Gy[−1.58−0.05]	0.11%[−0.22−0.55] 0.01 Gy[−0.01−0.04]	−0.56%[−2.02−0.65] −0.04 Gy[−0.16−0.04]	−0.34%[−2.02−2.56] −0.06 Gy[−0.34−0.04]

Abbreviations: CT, computed tomograpy; DVH, dose–volume histogram; MRI, magnetic resonance imaging; OAR, organ at risk; PRV, planning organ at risk at volume.

## 4 DISCUSSION

This study evaluated the effect of bone rED assignment on dose calculations in abdominal radiotherapy treatment planning using the bulk density method in patients with liver, pancreatic, and kidney cancer. The mean rED values for the 20 patients were 0.291 for the lungs and 0.994 for the body contour. These values were comparable to those referenced in the ICRU report (0.258 for lung, 1.000 for water, and 1.019 for soft tissue). However, abdominal organs that could contain air pockets (colon, small bowel, duodenum, and stomach) had mean rED values in the range of 0.840–0.983. This may be one of the reasons why Scenarios B and C, in which the rED value of the body was uniformly set to bulk density, showed larger dose differences than Scenario A. In this study, we examined CT‐based reference plans using voxel‐based rED information and MRI‐based plans using the bulk density method. CT‐based validation minimized the uncertainty of the bulk density method, enabling the evaluation of the impact of bone rED assignment in the abdominal region. Furthermore, MRI validation enabled assessment of the impact without bone rED assignment in treatment planning methods using the bulk density method implemented in current MR‐Linac workflows. In scenario A, the average gamma pass rate (2%/2 mm) exceeded 98% for CT and MRI validation, and all target DVH parameters were within a 1.0% difference. This suggests that while scenario A is generally acceptable for creating treatment plan in the abdominal region, the uncertainty between the bulk density method and voxel‐based dose calculation should be acknowledged. Hsu et al. reported that the dose difference between the population‐based bulk density method and the CT image was approximately −0.4%, even when only the body contours, bones, and lungs were segmented.^10^ In this study, the absolute mean difference between the CT‐based calculations and the bulk density method was 0.51%. Thus, our results were consistent with theirs in showing that dose calculations between CT images and the bulk density method included an uncertainty of approximately 0.5% in the DVH comparisons. Additionally, in the gamma analysis using RP_CT, the average gamma pass rate was approximately 96.3% between bulk density methods and the CT‐based dose calculation. Therefore, when applying the results of this study to treatment plans created with the bulk density method, these uncertainties should be taken into consideration. For DVH comparison, Korsholm et al. reported that MR‐only radiotherapy can be used reliably in clinical practice, as the dose difference compared to the calculations from CT imaging was <2%.^17^ For scenario A, where recalculations were performed without assigning rED to bones from the RP conditions in the CT and MRI datasets, in the DVH for targets, all DVH indices were within a 1.0% difference. In addition, the absolute dose differences of the OAR DVH parameters were no greater than 0.3 Gy. Additionally, the results of Scenario_bulkCT and ScenarioA_bulkCT indicated that when compared with CT‐based dose calculations, the bulk density method and the bulk density method without bone rED assignment produced no substantial differences in this dataset (gamma pass rate: 96.3% vs. 96.0%). In the present study, dose distributions showed minimal differences when only bone rED assignment in the abdominal region was omitted when compared with the reference conditions. The MRI‐based validation utilizes reference plans that are established based on the bulk density method. This enabled a direct comparison of dosimetric differences when adopting the ICRU‐based assignment methodology. While the average gamma pass rates exceeded 95% when comparing scenarios B and C with RP_MRIs, notable differences were observed in the DVH parameters. Scenario A showed no cases with differences exceeding 1%, whereas ICRU‐based assignment demonstrated cases with errors exceeding 2%. In the worst cases, dose differences were observed in medium‐ to low‐dose regions, particularly because of beams traversing from the opposite side of the tumor and through organs prone to air pockets. Therefore, the clinical implementation of Scenarios B and C may require the optimization of beam arrangements to avoid both opposite‐side beam entry and traversal through organs susceptible to air pockets.

The findings of the present study are as follows. (i) The target and OAR DVHs and dose distributions may be minimally affected by bone rED assignment regardless of its implementation. (ii) The effect of bone segmentation on obtaining an appropriate rED of the body contour from CT images is minimal in clinical practice, as the calculations obtained with and without bone exhibit a small difference (<0.1%). A major limitation of this study was the small number of patients with pancreatic cancer. Another limitation was the treatment of the spine and rib as a single bone. The impact of bone rED on the spine may be greater if beam arrangements frequently pass through the spine to reach the target, as observed in the treatment plans for patients with pancreatic cancer. Although the effect of the spine was minimal in the present subgroup analysis, further studies with a larger cohort of patients with pancreatic cancer and consideration of the beam angles may be necessary.

## 5 CONCLUSION

The present study evaluated the impact of bone rED assignment on radiotherapy planning in the abdominal region. Based on voxel‐based rED evaluation using CT datasets, the findings of the present study suggest that the assignment of bone rED values may have minimal impact on dose calculations in abdominal radiotherapy treatment planning. Furthermore, similar results may be applied to clinical MR‐Linac radiotherapy planning if the uncertainties associated with the bulk density method are adequately considered.

## AUTHOR CONTRIBUTIONS

Kota Abe contributed to the design of the study and mainly drafted the manuscript. Kota Abe and Yukinao Abe contributed to the data analysis. Masato Tsuneda and Yukio Fujita conceived the study and performed data analysis. Takashi Uno reviewed all the clinical treatment plans. All authors have read and approved the final manuscript.

## CONFLICT OF INTEREST STATEMENT

Kota Abe, Masato Tsuneda, and Yukio Fujita receive endowed chairs funded by Elekta K.K.

## Supporting information



Supporting information
