# Supplementary material for: Impact of bulk density assignment of bone on MRI‐based abdominal region radiotherapy planning for MR‐linac workflow
Source: J Appl Clin Med Phys. 2025 Feb 25;26(6):e70059. doi: 10.1002/acm2.70059 (PMC12148759; doi:10.1002/acm2.70059)
Supplement: Supplementary file 1 — Supporting information [file ACM2-26-e70059-s001.pdf]

Supplement materials

To demonstrate the reliability of the calculation method used in this study, we analyzed a case of a patient with spinal metastasis. In this case, the planning target volume (PTV) contour was used as a mask, and gamma analysis was performed within that region. Significant discrepancies in the targets and organ at risk (OAR) dose-volume histogram (DVH) parameters may occur if relative electron density (rED) assignment to the bone was not performed in this case. The bone rED value for this case was 1.133. The reference plan was established using CT image (RP_CT) with a voxel-based rED assignment method. For Scenario A, RP_CT was recalculated by overriding the bone rED with the body contour value (rED = 0.949). Using RP_CT as reference, the gamma analysis results for Scenario A dose distribution are shown in Figure S1. The relative dose differences for DVH parameters are presented in Table S1.The gamma pass rates were below 90%, and all DVH indices showed dose differences exceeding 2%.

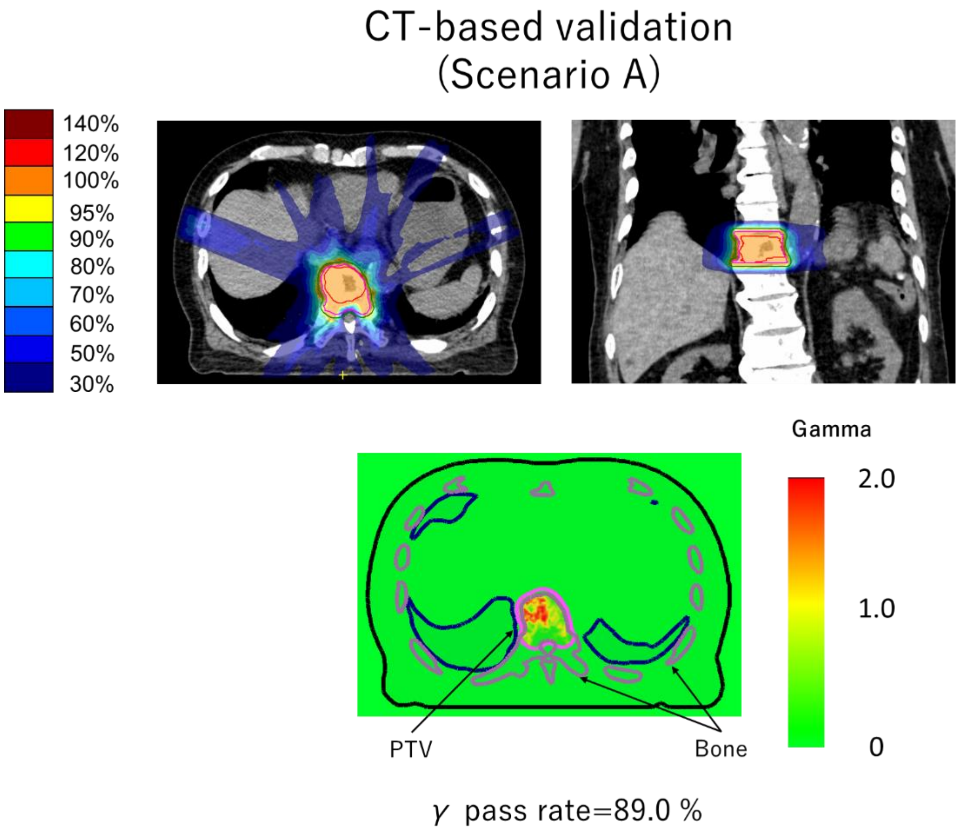

Fig S1. Gamma maps and gamma pass rate for scenario A of gamma analysis on CT image sets with reference plan (dose difference/distance to agreement = 2%/2mm) in patient with spinal bone metastasis. Gamma analysis was performed with the planning target volume (PTV) as a mask to evaluate dose distribution differences within the PTV.

Table S1. The relative percentage differences for scenario A compared with the reference plan on CT dataset for target and OAR DVH parameters in patient with spinal bone metastasis.

	Relative percentage difference [%]					
	GTV D95%	CTV D95%	PTV D95%	PTV D99%	PTV D1 cc	PRV spinal cord D0.035 cc
Scenario A (1 case)	2.70	2.81	2.50	5.13	2.49	4.98

Abbreviations: OAR, organ at risk; DVH, dose–volume histogram; GTV, gross tumor volume; CTV, clinical target volume; PTV, planning target volume; PRV, planning organ at risk at volume
